# Biological Effect of Different Spinach Extracts in Comparison with the Individual Components of the Phytocomplex

**DOI:** 10.3390/foods10020382

**Published:** 2021-02-09

**Authors:** Laura Arru, Francesca Mussi, Luca Forti, Annamaria Buschini

**Affiliations:** 1 Department of Life Sciences, University of Modena and Reggio Emilia, 41125 Modena, Italy; francesca.mussi@unipr.it (F.M.); luca.forti@unimore.it (L.F.); 2 International Center BIOGEST-SITEIA, University of Modena and Reggio Emilia, 42122 Reggio Emilia, Italy; 3 Department of Chemistry, Life Sciences and Environmental Sustainability, University of Parma, 43124 Parma, Italy; 4 International Center COMT, University of Parma, 43124 Parma, Italy

**Keywords:** phytocomplex, spinach extracts, colon cancer cell line, phytochemicals, antioxidants

## Abstract

The Mediterranean-style diet is rich in fruit and vegetables and has a great impact on the prevention of major chronic diseases, such as cardiovascular diseases and cancer. In this work we investigated the ability of spinach extracts obtained by different extraction methods and of the single main components of the phytocomplex, alone or mixed, to modulate proliferation, antioxidant defense, and genotoxicity of HT29 human colorectal cells. Spinach extracts show dose-dependent activity, increasing the level of intracellular endogenous reactive oxygen species (ROS) when tested at higher doses. In the presence of oxidative stress, the activity is related to the oxidizing agent involved (H_2_O_2_ or menadione) and by the extraction method. The single components of the phytocomplex, alone or mixed, do not alter the intracellular endogenous level of ROS but again, in the presence of an oxidative insult, the modulation of antioxidant defense depends on the oxidizing agent used. The application of the phytocomplex extracts seem to be more effective than the application of the single phytocomplex components.

## 1. Introduction

The lifestyle of the most industrialized countries brings many benefits but can induce potential risks that can worsen the quality of life. Sedentary lifestyle, improper nutrition, unbalanced diet, chaotic pace of today’s life, just to name a few, can have negative effects on human health; especially a fat-rich diet leads to oxidative stress which in turn can contribute to the onset of degenerative diseases [1].

There is increasing evidence of a close correlation between diet and risk of cancer, both in positive (prevention) and negative (development of the disease) sense [2]. The introduction of flavonoids, carotenoids, omega-3 fatty acids, vitamins, minerals, antioxidants through fruit and vegetables seems to have positive effects in reducing some types of cancer and chronic diseases, thanks to the ability of these molecules to reduce the damage caused by reactive oxygen species (ROS) [2,3,4].

Polyphenols, and antioxidants in general, act as free radical scavengers and metal chelators, helping the physiological cell response in counteracting the damage induced by ROS.

Intracellular ROS are normally generated during the cellular biochemical processes, and several cellular signaling pathways are regulated by ROS [5]. However, when their level happens to be increased by external agents (i.e., ionizing radiation, pollutants with chlorinated compounds or metal ions that may directly or indirectly generate ROS) they can damage proteins, lipids, and DNA, leading to impaired physiological functions with a decreased proliferative response, defective host defense and cell death [6].

Oxidative stress occurs when there is an imbalance between the intracellular levels of ROS and the cell defense systems; this can help the insurgence of diseases such as cardiovascular diseases, neurodegenerative diseases, and cancer [7]. Cells need to maintain the physiological homeostasis by balancing the ROS levels and the antioxidant defenses [6].

Not all antioxidant molecules have the same protective effect, but it is also known that the effect of a phytocomplex, a set of active ingredients contained in vegetable food, is often synergistic and greater than the effect that the single components can have. This can be in part explained because there is not a singular molecular target for a disease, but often disease is a result of a multi-factorial causality [8].

Furthermore, it should also be considered that the relative percentages of the constituents of the phytocomplex could play a decisive role in determining its effectiveness. Often, scientific attention focuses on a single active molecule, or on a few known constituents. However, the synergistic effect of the phytocomplex can be lost when testing single molecules or when, in the effort of extracting the phytocomplex, part of its minor components is lost [8,9,10,11].

Spinach (*Spinacia oleracea* L.) belongs to the family of Chenopodiaceae and it is a proven source of essential nutrients such as carotene (a precursor of vitamin A), ascorbic acid, and several types of minerals. According to the Agricultural Research Service of the U.S. Department of Agriculture, 100 g of fresh spinach provides at least 20% or more of the recommended dietary intake of β-carotene (provitamin A), lutein, folate (vitamin B9), α-tocopherol (vitamin E) and ascorbic acid (vitamin C). Moreover, spinach leaves contain flavonoids [12] and phenolic acids such as ferulic acid, ortho-coumaric and para-coumaric acids [13]. In 2009, Hait-Darshan and colleagues isolated from spinach leaves a mixture of antioxidants defined NAO (natural antioxidant) that contains aromatic polyphenols, including the phenolic acids and the derivatives of the glucuronic acid [14]. NAO can effectively counteract free radicals [15,16] resulting in an antiproliferative and anti-inflammatory potential, in vivo and in vitro [17].

In a previous study [18], we have already shown the ability of spinach leaf juice to inhibit the proliferation of the human HT29 colon cancer cell line in a time and dose-dependent manner. The juice significantly also reduced the damage induced by a known oxidant agent up to 80%.

In this study, we evaluated the biological effects of different spinach extracts (hydrophilic, liquid nitrogen, and water extraction) and of some of the main components, alone or mixed together, on the human colorectal adenocarcinoma HT29, which is a cell line representative of the gastrointestinal tract and a recognized good model for the study of the correlation between diet and carcinogenesis [19]. We chose this approach as a step forward our previous research [18]: the spinach juice from fresh leaves proved to have both anti-proliferative and antioxidant effect. This time we aimed (1) to investigate if different extraction methods could positively influence this outcome; (2) to test the effect of the main components of the phytocomplex previously identified, when submitted alone or mixed in a sort of artificial simplified phytocomplex.

## 2. Materials and Methods

### 2.1. Spinacia oleracea Extracts

In this study, three extraction methods have been used to obtain a phytocomplex rich in different polyphenols and other relevant biological active molecules fractions. To obtain an extract rich in hydrophilic compounds (hydrophilic extract, HE), water was added to spinach leaves in a 1:1 (*w/v*) ratio [15]. The leaves were ground in a mortar, filtered with sterile gauze, the liquid transferred into a micro-tube, and centrifuged twice for 12 min at 15,000× *g*. The collected supernatant was concentrated (Speed Vacuum Concentrator, Eppendorf 5301, Eppendorf, AG, Hamburg, Germany) to 25%, then added a 9:1 volume of acetone in water, vortexed for 5 min and centrifuged for 12 min at 15,000× *g*. The supernatant was carefully transferred to a fresh tube and completely dried. The dry extract was chilled on ice and stored at −20 °C until use. Water extract (WE) was obtained by simply adding sterile distilled water in a 1:1 ratio in the first step of the above-described procedure. Liquid nitrogen extract (NE) was obtained by grinding leaf samples in a mortar with liquid nitrogen, mashed material weighed, and transferred in a 10 mL syringe to be filtered with a 60 µm nylon filter. The extract was centrifuged for 10 min at 8000× *g* (4°C) and the supernatant was filtered (0.2 µm filter).

### 2.2. HT29 Cell Line

Cells were thawed and grown in tissue culture flasks as a monolayer in DMEM (Dulbecco’s Modified Eagle Medium), supplemented with 1% L-glutamine (2 mM), 1% penicillin (5000 U/mL)/streptomycin (5000 µg/mL) and 10% fetal bovine serum (FBS) at 37 °C in a humidified CO_2_ (5%) incubator. The cultured cells were trypsinized with trypsin/EDTA for a maximum of 8 min and seeded with a subcultivation ratio of 1:3–1:8. Determination of cell numbers and viabilities was performed with the trypan blue exclusion test.

### 2.3. Modulation of the Proliferation

#### 2.3.1. MTS Assay

To determine cell viability, in the exponential phase of the growth cells were seeded at 5 × 10^4^/mL in 96-well plates in medium supplemented with 1% glutamine, 0.5% penicillin/streptomycin, and 5% fetal bovine serum. After seeding (24 h), cells were treated, in quadruplicate, with increasing concentrations of phytochemicals (1–500 μM) and incubated for 24 and 48 h. Ascorbic acid, 20-hydroxyecdysone, ferulic acid, 2-hydroxycinnamic acid, *p*-coumaric acid, β-carotene, and lutein were from Sigma-Aldrich Company Ltd. (Milan, Italy) and resuspended in dimethylsulfoxide. The cytotoxicity assay was performed by adding a small amount of the CellTiter 96R AQueous One Solution Cell Proliferation Assay (Promega Corporation, Madison, WI, USA) directly to culture wells, incubating for 4 h and then recording the absorbance at 450 nm with a 96-well plate reader (MULTISKAN EX, Thermo Electron Corporation, Vantaa, Finland). The percentage of cell growth is calculated as:

growth % = 100 − [1 − (OD450 treated/OD450 untreated)] × 100(1)

#### 2.3.2. Trypan Blue Exclusion Method

Different concentrations of spinach extracts (1%, 5%, 10%, 50%) were added to the cells medium. Cells were seeded in 6-well plates (2 mL/well) at the density of 2 × 10^5^ cell/well. After 24 or 48 h of treatment, cells were trypsinized and resuspended in DMEM; a 1:1 dilution of the cell suspension was obtained using a 0.4% trypan blue solution (BioWhittake^®^, Lonza, Walkersville, MD, USA). The dilution was loaded on a counting chamber of a hemocytometer: since the dye freely passes only through the permeabilized membranes of dead cells, the percentage of viable cells can be evaluated. For each sample, 100 cells were scored.

#### 2.3.3. Comet Assay

Cells were seeded at 1 × 10^5^/mL in 6-well plates in DMEM supplemented with 1% glutamine, 0.5% penicillin/streptomycin, and 10% fetal bovine serum. After seeding (24 h), HT29 cells were treated with single phytochemicals, a mixture of them at the lower concentration, and spinach extracts (1%, 5%, 10%). The phytochemical concentration was chosen according to the amount that can be found in 100 g of fresh spinach. A concentration tenfold higher was also tested to evidence possible activity variation directly related to the concentration (Table 1). To assess possible synergic or antagonist effects, the activity of a mixture of the phytochemicals at the lower dosage was also evaluated. After 24 h of incubation at 37 °C, the cells were trypsinized and resuspended in DMEM at a concentration of 5 × 10^4^ cell/mL; centrifuged (1 min, 800× *g*) and the cell pellet resuspended in 90 µL Low Melting Agarose 0.7% (LMA), before being transferred onto degreased microscope slides previously dipped in 1% normal melting agarose (NMA) for the first layer. The agarose was allowed to set for 15 min at 4 °C before the addition of a final layer of LMA. Cell lysis was carried out at 4 °C overnight in lysis buffer (2.5 M NaCl, 100 mM Na2EDTA, 8 mM Tris-HCl, 1% Triton X-100, and 10% DMSO, pH 10). The electrophoretic migration was performed in alkaline buffer (1 mM Na_2_EDTA, 300 mM NaOH, 0 °C) at pH > 13 (DNA unwinding: 20 min; electrophoresis: 20 min, 0.78 Vcm^−1^, 300 mA). DNA was stained with 75 µL ethidium bromide (10 µg/mL) before the examination at 400× *g* magnification under a Leica DMLS fluorescence microscope (excitation filter BP 515–560 nm, barrier filter LP 580 nm), using an automatic image analysis system (Comet Assay III Perceptive Instruments Ltd., Bury St Edmunds, UK). Total fluorescence % in tail (TI, tail intensity) provided representative data on genotoxic effects. For each sample, coded and evaluated blind, at least three independent experiments were performed, 100 cells were analyzed, and the median value of TI was calculated.

#### 2.3.4. Comet Assay—Antioxidant Activity

Cells were seeded and incubated with phytochemicals/extracts as described above. After incubation and trypsinization, cells were resuspended in DMEM (supplemented with 1% glutamine, 0.5% penicillin/streptomycin and 10% fetal bovine serum) at a concentration 1 × 10^5^ cell/mL for further treatment in suspension before to perform the Comet assay, with H_2_O_2_ (100 μM) on ice for 5 min. The suspensions were then centrifuged twice (1 min, 800× *g*) to wash and recover the cells. The slides were prepared and analyzed as reported above.

### 2.4. Measurement of Reactive Oxygen Species (ROS) Production

Cells were seeded at 1 × 10^5^/mL in 24-well plates in DMEM supplemented as described above. After seeding (24 h), cells were treated for 24 h with ascorbic acid 3 and 30 μM, 20-hydroxyecdysone 1.5 and 15 μM, ferulic acid 1 and 10 μM, 2-hydroxycinnamic acid 2 and 20 μM, *p*-coumaric acid 0.1 and 1 μM, β-carotene 1.5 and 15 μM, lutein 2 and 20 μM, with the phytochemicals mixture (at their lowest concentration) and with spinach extracts at 1–5–10%. After 24 h of treatment, cells were washed with PBS and pre-incubated for 30 min (37 °C) in the dark with DCFH-DA 10 μM diluted in PBS (pH 7.4). Cells were washed with PBS to remove extracellular DCFH-DA, resuspended in DMEM, and treated 30 min at 37 °C with menadione 100 μM, a known oxidant agent [20]. The medium was removed and a lysis solution (Tris-HCL 50 mM, 0.5% TritonX pH 7.4; cell dissociation solution, Sigma Aldrich, St. Louis, MO, USA) was added for 10 min. Cell lysates were scraped from the dishes and the extracts were centrifuged. The supernatant was collected, and the fluorescence was read with a fluorescence spectrophotometer (Spectra Fluor Plus, Tecan Group Ltd., Männedorf, Switzerland) looking at the fluorescence peak between 510 and 550 nm. Each experiment was performed in triplicate.

### 2.5. Statistical Analysis

Data were analyzed by univariate analysis of variance (ANOVA) with the Bonferroni multiple comparison post-hoc test through the SPSS 18.0 software (SPSS Inc., Chicago, IL, USA). For each experiment, performed in triplicate, the significance was accepted for *p* < 0.05.

## 3. Results

### 3.1. Modulation of the Proliferation

#### 3.1.1. MTS Assay

**Figure 1:**
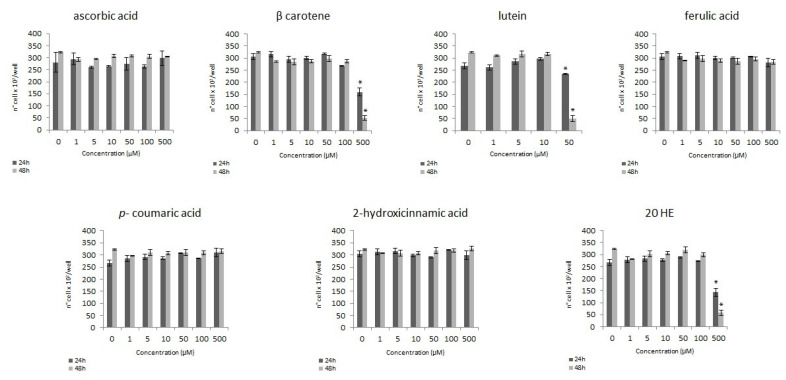
Modulation of the proliferation of HT29 cell line: number of cells/well on concentration after 24 and 48 h of treatment with increasing phytochemical concentration (* *p* < 0.05).

This colorimetric assay allows to evaluate if and to what extent the single phytochemicals tested can affect the proliferation of the HT29 cells, quantifying the number of cells in active proliferation. After 24 h and 48 h of treatment with increasing concentrations (1–500 µM) of ascorbic acid and hydroxycinnamic acids (ferulic-, *p*-coumaric- and 2-hydroxycinnamic acid) no variations have been found in the number of viable cells comparing treated and untreated samples (Figure 1). However, a significant decrease of cell proliferation has been recorded at the higher concentration tested (500 µM) after treatment with β-carotene, 20-hydroxyecdysone and lutein (Figure 1).

#### 3.1.2. Trypan Blue Exclusion Method

The MTS assay is not recommended for testing cell viability to spinach extract, since the presence of fibers and debris can interfere with the assay. In this case, the trypan blue exclusion method has been chosen to evaluate the modulation of cell proliferation.

After 24 and 48 h of treatment with increasing concentration (1%, 5%, 10%, 50%) of water (WE) and liquid nitrogen (NE) extracts, data indicate an antiproliferative activity related to highest concentrations.

**Figure 2:**
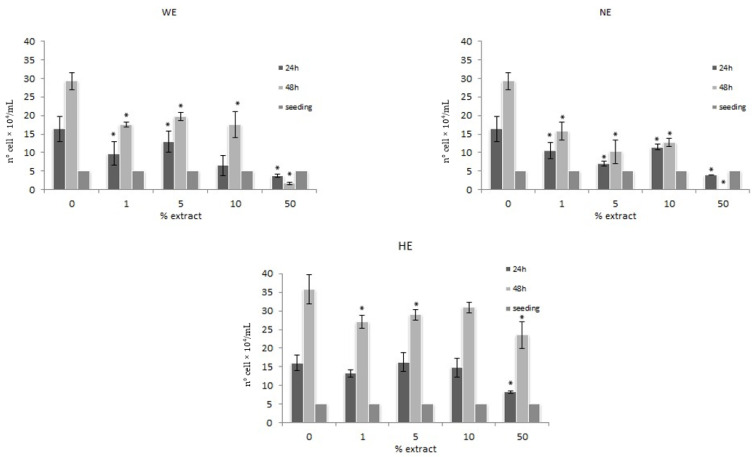
Modulation of the proliferation of HT29 cell line: number of cells/mL after 24 (dark grey) and 48 (light grey) hours of treatment with increasing concentrations of spinach extract. WE = water extract, NE = liquid nitrogen extract, HE = hydrophilic extract (* *p* < 0.05 taking into account the growth at 0 concentration). Seeding is the number of cells at time 0.

The hydrophilic extract (HE), at 48 h of treatment, shows a dose-dependent inhibition of proliferation and the highest concentration tested (50%) not only induces a reduction in cell division but also a strong cytotoxic effect. (Figure 2).

### 3.2. Genotoxic Activity

A Comet assay has been carried out to evaluate if the single phytochemicals, their mixture, or the spinach extracts exert a genotoxic effect on the HT29 cell line. This assay considers the onset of possible DNA damage by evaluating the presence, after electrophoresis, of fragmented DNA outside the core of the cell nucleus. Each phytochemical was tested considering the quantity that can be found in 100 g of fresh spinach as approximate mean in standard growth conditions (considering that cultivar, production method, and growing season can all impact on the nutrient composition), and at a concentration tenfold higher (Table 1); the mixture was prepared considering the lower concentration; the extracts were tested in the concentrations of 1%, 5%, and 10%.

**Figure 3:**
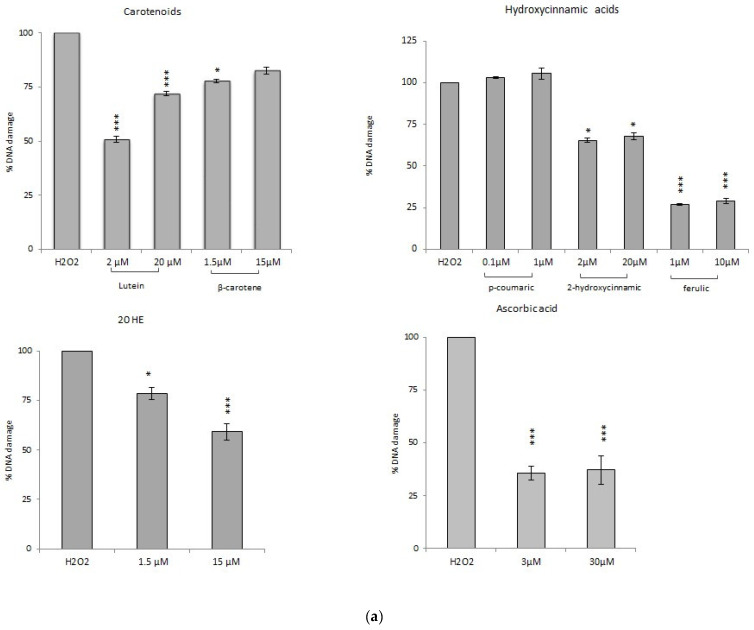

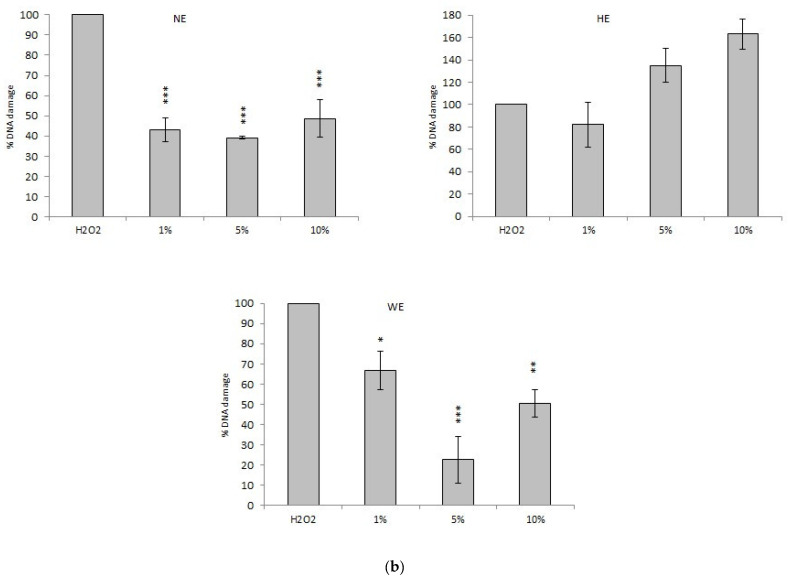
Percentage of reduction of DNA damage induced by H_2_O_2_ after a 24 h pre-treatment with different concentration (* *p* < 0.05; ** *p* < 0.01; *** *p* < 0.001): (**a**) pure phytochemicals; (**b**) extracts.

After 24 h of treatment with the single phytochemicals, no genotoxic effect was observed except for lutein 20 µM that showed a significant increase in tail intensity (TI, Table 2). This could explain the antiproliferative activity observed at this concentration with the MTS assay (Figure 1), related to DNA damage somehow induced by lutein. The treatment with the mixture of phytochemicals does not lead to any observed genotoxic effect as well (Table 2). Apart WE, the 24 h treatment with NE and HE led to genotoxic effect when tested at higher concentration (Table 2). This behavior could be partially responsible of the results reported in the Comet assay after oxidative injury (Figure 3).

### 3.3. Antioxidant Activity

#### 3.3.1. Comet Assay

The antioxidant activity was evaluated as the ability of the samples to increase the endocellular defenses against an external oxidative stress. The Comet assay was carried out after 24 h of treatment to measure the protection against oxidative DNA damage induced by H_2_O_2_ (100 µM).

##### Comet Assay with Phytochemicals

The chemicals that showed non-antiproliferative activity also exhibit a significant DNA damage reduction after H_2_O_2_ oxidative stress: up to 60% for ascorbic acid (3 and 30 µM) and 75% for ferulic acid (1 and 10 µM) without variations between concentrations tested. 2-hydroxycinnamic acid gives a DNA damage reduction of about 25% (2 and 20 µM). Only *p*-coumaric acid does not show any antioxidant activity; this might be related to the very low concentrations tested (0.1 and 1 µM) or to the fact that the molecule, acting as a scavenger, may have another main target (i.e., superoxide anion) (Figure 3).

Among the antiproliferative phytochemicals, DNA damage reduction up to 40% was recorded with the highest dose of 20-hydroxyecdysone (1.5 and 15 µM) and, among the carotenoids, up to 50% with lutein 2 µM. β-carotene (1.5 and 15 µM) reduces DNA damage of about 15–20%. Despite its genotoxicity, also lutein 20 µM seems to induce a certain level of protection (~30%); the same level (~32%) observed after pre-treatment with the mixture of phytochemicals (Figure 3).

##### Comet Assay with Spinach Extracts

All the extracts have been tested at 1%, 5%, and 10%. The WE shows a significant dose-dependent reduction of DNA damage: the 5% concentration seems to be the most active, with a DNA damage reduction of about 78%. The NE significantly reduces DNA damage up to 60%, without differences of activity among the concentrations tested. HE does not show the ability to counteract the damage induced by hydrogen peroxide: on the contrary, at the highest dosage, it shows a pro-oxidant activity (Figure 3).

#### 3.3.2. Measurement of Variation in Reactive Oxygen Species (ROS) Concentration

The samples’ ability to counteract the increase of ROS induced by an oxidizing agent has been investigated. For this purpose, menadione (vitamin K3) was used, a synthetic derivative of the natural vitamins K1 and K2 with a degree of toxicity against a wide variety of cancer cells. It can act directly, through the formation of reactive oxygen species, or indirectly through the depletion of the most important endogenous antioxidant, glutathione (GSH). The level of ROS was measured by a fluorescence assay with 2′,7′-dichlorofluorescein-diacetate (DCFH-DA), a non-fluorescent compound that crosses cell membranes. Once in the cytoplasm, esterases remove the acetates to produce 2′,7′-dichlorofluorescein (DCFH), which is not cell permeable anymore. DCFH is easily oxidized to 2′,7′-dichlorofluorescein (DCF), a highly fluorescent compound.

**Figure 4:**
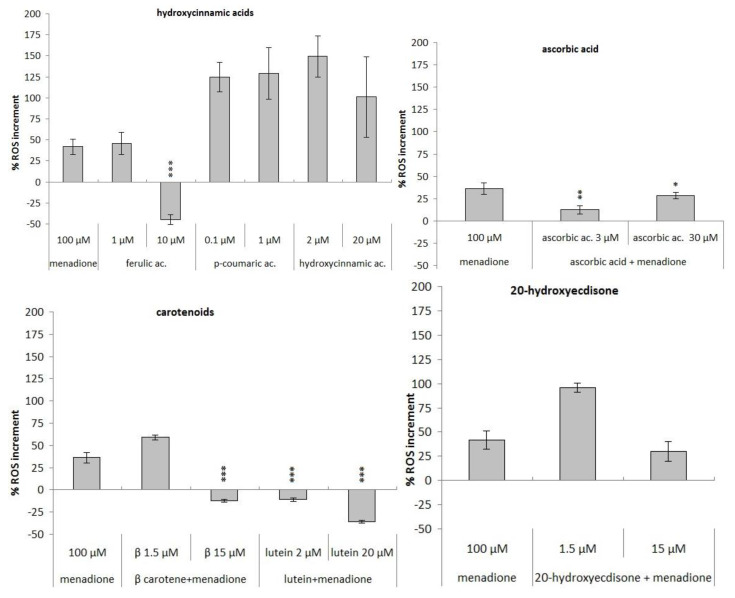
Evaluation of antioxidant ability of the phytochemicals to counteract the intracellular ROS levels caused by 24 h treatment with menadione oxidative insult (100 µM) on HT29 cell line, expressed as ROS increment % (* *p* < 0.05; ** *p* < 0.01; *** *p* < 0.001).

**Figure 5:**
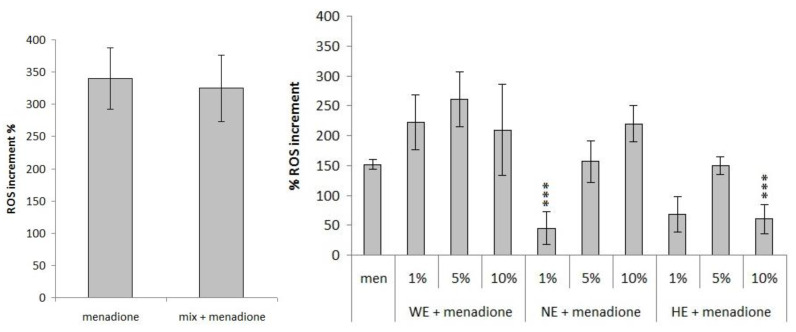
Evaluation of different spinach extract (left) and of single component mix (right) antioxidant ability to counteract menadione (100 µM) oxidative insult after 24 h of treatment expressed as ROS increment % (*** *p* < 0.001).

Only ascorbic acid, ferulic acid 10 µM, β-carotene at the highest dose and lutein can significantly inhibit the production of ROS, while 15 µM of 20-hydroxyecdysone weakly counteracts the increase of ROS levels induced by menadione (Figure 4). Interestingly, the synthetic mixture of the major components of the spinach complex presents no antioxidant activity, showing the presence of antagonistic effects (Figure 5). Among the extracts, only the lowest concentration of the liquid nitrogen extract (1%) and the highest concentration of the hydrophilic extract (10%) show the ability to significantly counteract the oxidative stress induced by this oxidizing agent (Figure 5).

## 4. Discussion

*Spinacia oleracea* has a good antioxidant activity related to the presence of a pool of known phytochemicals such as ascorbic acid, carotenoids (β-carotene and lutein), hydroxycinnamic acids (ferulic acid, *p*-coumaric acid, 2-hydroxycinnamic acid), and 20-hydroxyecdysone [18]. The mechanism of action of these phytochemicals is not yet fully understood, even if it seems to be related both to their concentrations and the different types of cell lines involved. In this work, the ability has been evaluated of different extracts to modulate the proliferation of the human adenocarcinoma cell line (HT29). It has also tested the effect of the main phytochemicals belonging to the *Spinacia oleracea* phytocomplex as previously identified [18], submitted alone or merged together, in a molarity calculated as an approximate mean of concentration present in a standard condition of 100 g of fresh spinach (Table 1), considering that cultivar, production method, and growing season can all impact on nutrient composition. The activity of the phytochemicals seems to be related to the concentration applied, and on how the oxidative stress has been induced on the cells. In addition, in the case of the spinach extracts, the biological activity also reflects the method of extraction used, suggesting a greater level of complexity.

Considering the single antioxidants tested, hydroxycinnamic acids seem not to have antiproliferative activity on this cell line (Figure 1), independently from the concentration (1–500 µM) or the length time of the treatment (24 h or 48 h), in agreement also with Martini et al. [21]. The same considerations can be made for ascorbic acid, which does not induce any change in HT29 cells viability in the tested concentrations range (Figure 1), according to what observed by Fernandes et al. in 2017 on bone cancer cells cell line [22].

On the other hand, treating the cells with β-carotene (500 µM) leads to a severe inhibition of the proliferation (Figure 1). Similar results were obtained by Park et al. [23] on gastric cancer cells. Upadhyaya [24] reported a reduction of proliferation after 12 h of treatment with β-carotene starting from a concentration of 20 μM on the leukemic cell lines U937 and HL60. Compared to them, the HT29 cell line seems to be less sensitive to the effect of β-carotene, suggesting different effects of the same molecule on various cell lines. Different effects can also be found observing the response to lutein: according to the results presented in this paper, the HT29 cell line seems to be less sensitive (Figure 1) than the human breast cancer cell lines MCF7 and MDA-MB-157, as it undergoes a significant concentration-dependent reduction of viability after 24 h of treatment with lutein 5–120 μM [25].

On the other hand, all the spinach extracts demonstrate an antiproliferative activity on the HT29 cell line regardless of the type of extraction (Figure 2). While the HE shows dose-dependent inhibition of the proliferation, WE and NE show both time and dose-dependent effect, with a cytotoxic activity only at the highest concentrations tested. These results agree with what was observed by [26] on the colon cancer cell line Caco2 treated with *Amaranthus gangeticus* L. (red spinach) aqueous extracts and by Fornaciari et al. [18] on the HT29 cell line treated with *Spinacia oleracea* extracts.

Besides the antiproliferative activity, also the possible genotoxic effect of the single phytochemicals, of their mixture, and of the different spinach extracts have been evaluated by Comet assay, a useful approach to study the effect of nutrients and micronutrients. The data obtained with the hydroxycinnamic acids integrate what was already been reported by Ferguson et al. [27], namely the absence of genotoxicity at concentrations (0.5 and 1 mM) and time of treatment (7 days) higher than those assessed in the present article. Regarding the dose-dependent genotoxicity of lutein, similar behavior has been reported by Kalariya and colleagues [28]: after 9 h of treatment with lutein metabolites starting from the concentration 10 μM, a significant increment in tail intensity was observed on the retinal pigmented epithelium (ARPE-19). The presence of a genotoxic effect at 20 µM may suggest the involvement of a direct action of lutein on DNA, resulting in the reduction of the proliferation previously observed at concentrations higher than 10 µM.

The protective effect of foods rich in antioxidants carried out as decreased sensitivity to the damage induced by known oxidizing agents has been widely demonstrated [2,29]. In this work, we compared the antioxidant activity of extracts and chemicals using a modified protocol of the Comet assay that allows to verify the ability to reduce the extent of the DNA damage induced by hydrogen peroxide.

The phytochemicals mixture shows a significant DNA damage reduction, by 32% (Figure 3). However, it seems that there is not an additive antioxidant effect when the single phytochemicals are mixed, since the single molecules reached higher percentages of DNA damage reduction. Comparing the mixture to the natural phytocomplex, WE and NE show a significantly higher DNA damage reduction up to 75% (Figure 3). A similar result was observed by Ko and colleagues in 2014 on HepG2 cells and human leukocytes treated with *Spinacia oleracea* water extracts [1]. The activity of NE seems to be independent by its concentration, with a persistent reduction near 60%. WE shows a dose-dependent activity at the lowest doses with a DNA damage reduction up to 75%, while it loses effectiveness at the highest concentration. HE does not show activity at the lowest dose, while it proves a pro-oxidant effect at the highest ones. These observations suggest that the method of extraction strongly influences the molecular content of the phytocomplex and therefore the biological activity of the resulting extract. The WE activity recalls what has been observed for ascorbic acid (and other antioxidant molecules), high concentrations minimize the antioxidant effect or induce a pro-oxidant effect [30]. The antioxidant activity was further investigated by measuring the ability of the samples to modulate the HT29 physiological levels of ROS. Among the phytochemicals, only the highest concentration of ferulic acid, lutein, and 20-hydroxyecdysone significantly increases the intracellular ROS levels disturbing the cell oxidative balance (data not shown). A similar pro-oxidant effect was reported for ferulic acid in two cervical cancer cell lines (HeLa and ME-180) by Karthikeyan et al. in 2011 [31]. The induced imbalance does not seem to alter cell proliferation except for the treatment with lutein that, at the higher concentration, can increase the intracellular ROS levels, to inhibit cell proliferation and to induce significant DNA damage. The phytochemicals mixture does not induce ROS level variations, according to the behavior of the single molecules (Figure 5). The spinach extracts show an alteration of the intracellular ROS levels only at the higher tested concentration (10%), with a significant increase of ROS (Figure 5). Only NE shows a similar pro-oxidant effect already at a lower assayed concentration (5%).

Thereafter, also, the ability of the samples to counteract the increase of ROS induced by menadione has been evaluated. Among the single phytochemicals, β-carotene 15 µM, lutein 2 µM, and ascorbic acid 3 and 30 µM (which do not induce significant variations of intracellular ROS levels in physiological conditions, data not shown) significantly counteract the activity of menadione (Figure 4). The 10 µM ferulic acid and the 20 µM lutein induce an increase of ROS levels in the absence of stress and strongly reduce them in the presence of stress (Figure 4). The mixture of phytochemicals does not induce any variation of ROS levels (Figure 5); it seems that the simultaneous presence of all the phytochemicals can interfere with their activity. The different extracts action, able to exhibit pro-oxidant effect both in the absence and in the presence of oxidative stress and to significantly counteract the increase due to menadione, points out again conflicting results depending on the method of detection and on the oxidant agent used.

The *p*-coumaric acid alone does not reduce the oxidative damage induced either by hydrogen peroxide or by menadione. The *p*-coumaric acid normally acts as a scavenger of superoxide anions; this could explain its inability to counteract hydrogen peroxide. Moreover, since the *p*-coumaric acid is unable to counteract the effect of menadione, which can act reducing the levels of glutathione, it can be supposed that the antioxidant activity of the *p*-coumaric acid is closely associated with that of glutathione.

The 2-hydroxycinnamic acid does not alter the intracellular levels of ROS in physiological conditions, while, in the case of oxidative stress, its activity seems to be related to the type of oxidant agent involved: a pro-oxidant effect was observed in presence of menadione, suggesting an activity comparable to that of the *p*-coumaric acid; an antioxidant effect was observed in the presence of hydrogen peroxide, suggesting a scavenger activity towards a wider range of ROS.

In physiological conditions, the ferulic acid shows a dose-dependent activity, with a pro-oxidant effect at the highest dose. In case of stress, the ferulic acid has proved a dose-dependent antioxidant activity towards menadione and a greater dose-independent one towards the hydrogen peroxide. These observations suggest that the ferulic acid could act only directly in the presence of menadione, since it may interfere with the antioxidant endogenous systems. Moreover, the increased antioxidant activity showed towards the hydrogen peroxide, suggests that the ferulic acid can counteract this oxidative damage through a combination of a direct and indirect antioxidant activity.

In the absence of oxidative stress, 20 HE shows a dose-dependent activity, with a pro-oxidant effect at the highest concentration. In presence of stress, it shows a good antioxidant activity towards hydrogen peroxide and a dose-dependent activity towards menadione, counteracting it only at the highest dose. The β-carotene does not alter the intracellular levels of ROS in physiological conditions, while in the presence of stress it has shown a dose-independent antioxidant activity towards hydrogen peroxide and a dose-dependent activity towards menadione. Lutein counteracts both oxidant agents, even though in absence of stress it showed a pro-oxidant effect. The ascorbic acid does not induce alterations in the intracellular ROS levels in physiological conditions and it has shown significant antioxidant activity with both hydrogen peroxide and menadione.

In physiological conditions, the spinach extracts show a dose-dependent activity, increasing the intracellular ROS levels only at the highest doses. In the presence of oxidative stress, their activity is strongly related to the oxidant agent involved and to the method of extraction used. It is interesting to compare the biological activity of the single phytochemicals at the concentration that we can find in 100 g of fresh spinach, the “artificial” mixture of them, and the different spinach extracts at the lowest dose (1%), which corresponds to the available concentration coming from 100 g of fresh spinach. In physiological conditions, neither the single phytochemicals, nor the mixture, nor the spinach extracts induce alterations of the oxidative balance. In the presence of oxidative stress, a different behavior has been observed that is related to the kind of oxidative agent used. Almost all the single phytochemicals can reduce the oxidative damage induced by hydrogen peroxide, but when they are mixed there is not an additive antioxidant effect, since the single molecules reached higher percentages of DNA damage reduction. Considering the activity of the spinach extracts, they showed different activity towards the hydrogen peroxide depending on the method of extraction used and, consequently, on the bioactive molecules extracted. While the WE provides the same DNA damage reduction of the “artificial” mixture of phytochemicals, the NE shows improved effectiveness suggesting a synergic effect of the mixture of bioactive constituents contained, as often reported in the literature [8,9,32].

Against the oxidative damage induced by menadione, there is a more variable response. Among the single phytochemicals, only lutein and ascorbic acid are able to counteract the ROS levels increase, but when mixed with the other molecules they lose their ability. The spinach extracts also in this case show a different behavior depending on the method of extraction used: the HE is unable to counteract menadione, the WE shows a pro-oxidant effect and only NE reduces the intracellular ROS levels. Once again, it can be assumed that the biological effects of a plant extract are strongly related to a synergic effect of the bioactive molecules contained. A schematic overview of the activities described above is summarized in the following tables (Table 3 and Table 4):

Another interesting aspect that comes out from this work is the complex relationship between inhibition of the proliferation and the antioxidant activity. Considering the oxidative balance, any variation in the amount of intracellular reactive oxygen species can induce impaired physiological functions and a ROS reduction seems to be related to a decreased proliferative response, but the relation is not so direct. With almost all the compounds at low concentration an antioxidant activity can be noted but no inhibition of the proliferation. This latter activity is observed just for 20-hydroxyecdysone and β-carotene but at high concentrations (100 µM). Similar behavior is observed with lutein but the antiproliferative concentration, in this case, is lower than the biologically active one of the other compounds.

## 5. Conclusions

In this work, the biological activity of several phytochemicals applied to a human cell line at different concentrations was evaluated. The results lead to a confirmation of their beneficial properties, acting on ROS and lowering the oxidative damage. This highlights, among other possibilities, also that of considering the development of supplements including compounds derived from spinach extract, in order to defend health and to trigger possible anticancer effects.

## Figures and Tables

**Table 1:** Concentration of pure phytochemicals tested in comet assay.

Samples	Content (mg/100 g)	Assayed Concentrations	
Ascorbic acid	4.0	3 µM	30 µM
20-Hydroxyecdysone	5.0	1.5 µM	15 µM
Lutein	7.2	2 µM	20 µM
β-Carotene	5.1	1.5 µM	15 µM
Ferulic acid	1.0	1 µM	10 µM
*p*-Coumaric acid	0.1	0.1 µM	1 µM
2-Hydroxycinnamic acid	2.8	2 µM	20 µM

**Table 2:** Genotoxic effects of the different phytochemicals and extracts.

Samples	Concentration	TI% ^1^	Sd
NT ^2^		0.29	0.22
Lutein	2 μM	0.33	0.01
	20 μM	52.72	0.41
β-Carotene	1.5 μM	0.22	0.01
	15 μM	0.25	0.29
20HE	1.5 μM	0.18	0.01
	15 μM	0.59	0.23
Ascorbic acid	3 μM	0.32	0.35
	30 μM	0.25	0.29
*p*-Coumaric acid	0.1 μM	0.31	0.58
	1 μM	0.51	0.45
2-Hydroxicinnamic acid	2 μM	0.20	0.34
	20 μM	0.39	0.31
Ferulic acid	1 μM	0.21	0.14
	10 μM	0.20	0.07
Mix		0.23	0.11
WE	1%	0.69	0.17
	5%	1.03	0.01
	10%	0.77	0.06
	50%	4.33	0.70
NE	1%	0.86	0.08
	5%	0.38	0.08
	10%	1.33	0.46
	50%	toxic	
HE	1%	0.87	0.17
	5%	1.85	0.86
	10%	1.90	0.6
	50%	toxic	

^1^ Total fluorescence % in tail. ^2^ Not treated.

**Table 3:** Schematic overview of the antioxidant activity of the single phytochemicals and of their mixture (⇿ = no activity; ↑ = increase of oxidative stress; ↓ = reduction of oxidative stress).

Phytochemical (Physiological Dose)	Antioxidant Activity		
	No Stress	Menadione	H_2_O_2_
*p*-Coumaric acid	⇿	↑	⇿
2-Hydroxycinnamic acid	⇿	↑	↓ (40%)
Ferulic acid	⇿	↑	↓ (75%)
20 HE	⇿	↑	↓ (20%)
Ascorbic acid	⇿	↓	↓ (60%)
Lutein	⇿	↓	↓ (50%)
β-Carotene	⇿	⇿	↓ (20%)
Mix	⇿	⇿	↓ (32%)

**Table 4:** Schematic overview of the antioxidant activity of the phytochemical mixture and of the spinach extracts (⇿ = no activity; ↑ = increase of oxidative stress; ↓ = reduction of oxidative stress).

Sample	Antioxidant Activity		
	No Stress	Menadione	H_2_O_2_
Phytochemical mix	⇿	⇿	↓ (32%)
HE 1%	⇿	⇿	⇿
WE 1%	⇿	↑	↓ (35%)
NE 1%	⇿	↓	↓ (60%)

## Data Availability

The remaining data are available on request from the corresponding author.

## References

[1] Ko S.H., Park J.H., Kim S.Y., Lee S.W., Chun S.S., Park E. (2014). Antioxidant effects of spinach (*Spinacia oleracea* L.) supplementation in hyperlipidemic rats. Prev. Nutr. Food Sci..

[2] Parohan M., Anjom-Shoae J., Nasiri M., Khodadost M., Reza Khatibi S., Sadeghi O. (2019). Dietary total antioxidant capacity and mortality from all causes, cardiovascular disease and cancer: A systematic review and dose–response meta-analysis of prospective cohort studies. Eur. J. Nutr..

[3] Rajoria A., Kumar J., Chauhan A.K. (2010). Anti-oxidative and anti-carcinoginic role of lycopene in human health-A review. J. Dairy Foods Home Sci..

[4] Singh R.B., Hristova K., Fedacko J., Singhal S., Khan S., Wilson D.W., Takahashi T., Sharma Z. (2015). Antioxidant vitamins and oxidative stress in chronic heart failure. World Heart J..

[5] Ray P.D., Huang B.W., Tsuji Y. (2012). Reactive oxygen species (ROS) homeostasis and redox regulation in cellular signaling. Cell Signal..

[6] Valko M., Rhodes C.J., Moncol J., Izakovic M.M., Mazur M. (2006). Free radicals, metals and antioxidants in oxidative stress-induced cancer. Chem. Biol. Interact..

[7] Waris G., Ahsan H. (2006). Reactive oxygen species: Role in the development of cancer and various chronic conditions. J. Carcinog..

[8] Caesar L.K., Cech N.B. (2019). Synergy and antagonism in natural product extracts: When 1 + 1 does not equal 2. Nat. Prod. Rep..

[9] Wagner H., Ulrich-Merzenich G. (2009). Synergy research: Approaching a new generation of phytopharmaceuticals. Phytomedicine.

[10] Ulrich-Merzenich G., Panek D., Zeitler H., Vetter H., Wagner H. (2010). Drug development from natural products: Exploiting synergistic effects. Indian J. Exp. Biol..

[11] Junio H.A., Sy-Cordero A.A., Ettefagh K.A., Burns J.T., Micko K.T., Graf T.N., Richter S.J., Cannon R.E., Oberlies N.H., Cech N.B.J. (2011). Synergy-Directed Fractionation of Botanical Medicines: A Case Study with Goldenseal (*Hydrastis canadensis*). Nat. Prod..

[12] Gil M.I., Ferreres F., Tomas-Barberan F. (1999). Effect of postharvest storage and processing on the antioxidant constituents (flavonoids and vitamin C) of fresh-cut spinach. J. Agric. Food Chem..

[13] Bunea A., Andjelkovic M., Socaciu C., Bobis O., Neacsu M., Verhé R., Van Camp J. (2008). Total and individual carotenoids and phenolic acids content in fresh, refrigerated and processed spinach (*Spinacia oleracea* L.). Food Chem..

[14] Hait-Darshan R., Grossman S., Bergman M., Deutsch M., Zurgil N. (2009). Synergistic activity between a spinach-derived natural antioxidant (NAO) and commercial antioxidants in a variety of oxidation systems. Int. Food Res..

[15] Bergman M., Varshavsky L., Gottlieb H.E., Grossman S. (2001). The antioxidant activity of aqueous spinach extract: Chemical identification of active fractions. Phytochemistry.

[16] Lomnitski L., Bergman M., Nyska A., Ben-Shaul V., Grossman S. (2003). Composition, efficacy and safety of spinach extracts. Nutr. Cancer.

[17] Lomnitski L., Carbonatto M., Ben-Shaul V., Peano S., Conz A., Corradin L., Maronpot R.R., Grossman S., Nyska A. (2000). The prophylactic effects of natural water-soluble antioxidant from spinach and apocynin in a rabbit model of lipopolysaccharide-induced endotoxemia. Toxicol. Pathol..

[18] Fornaciari S., Milano F., Mussi F., Pinto-Sanchez L., Forti L., Buschini A., Arru L. (2015). Assessment of antioxidant and antiproliferative properties of spinach plants grown under low oxygen availability. J. Sci. Food Agric..

[19] Grajek W., Olejnik A. (2004). Epithelial cell cultures in vitro as a model to study functional properties of food. Pol. J. Food Nutr. Sci..

[20] Maioli E., Greci L., Soucek K., Hyzdalova M., Pecorelli A., Fortino V., Valacchi G. (2009). Rottlerin inhibits ROS formation and prevents NFkappaB activation in MCF-7 and HT-29 cells. J. Biomed. Biotechnol..

[21] Martini S., Conte A., Tagliazucchi D. (2019). Antiproliferative Activity and Cell Metabolism of Hydroxycinnamic Acids in Human Colon Adenocarcinoma Cell Lines. J. Agric. Food Chem..

[22] Fernandes G., Barone A.W., Dziak R. (2017). The effect of ascorbic acid on bone cancer cells *in vitro*. Cogent Biol..

[23] Park Y., Choi J., Lim J.W., Kim H. (2015). β-Carotene-induced apoptosis is mediated with loss of Ku proteins in gastric cancer AGS cells. Genes Nutr..

[24] Upadhyaya K.R., Radha K.S., Madhyastha H.K. (2007). Cell cycle regulation and induction of apoptosis by beta-carotene in U937 and HL-60 leukemia cells. J. Biochem. Mol. Biol..

[25] Li Y., Zhang Y., Liu X., Wang M., Wang P., Yang J., Zhang S. (2018). Lutein inhibits proliferation, invasion and migration of hypoxic breast cancer cells via downregulation of HES1. Int. J. Oncol..

[26] Sani H.A., Rahmat A., Ismail M., Rosli R., Endrini S. (2004). Potential anticancer effect of red spinach (*Amaranthus gangeticus*) extract. Asia Pac. J. Clin. Nutr..

[27] Ferguson L.R., Zhu S.T., Harris P.J. (2005). Antioxidant and antigenotoxic effects of plant cell wall hydroxycinnamic acids in cultured HT-29 cells. Mol. Nutr. Food Res..

[28] Kalariya N.M., Ramana K.V., Srivastava S.K., Van Kuijk F.J. (2008). Carotenoid derived aldehydes-induced oxidative stress causes apoptotic cell death in human retinal pigment epithelial cells. Exp. Eye Res..

[29] Griffiths K., Aggarwal B., Singh R., Buttar H., Wilson D., De Meester F. (2016). Food Antioxidants and Their Anti-Inflammatory Properties: A Potential Role in Cardiovascular Diseases and Cancer Prevention. Diseases.

[30] Milano F., Mussi F., Fornaciari S., Altunoz M., Forti L., Arru L., Buschini A. (2019). Oxygen Availability during Growth Modulates the Phytochemical Profile and the Chemo-Protective Properties of Spinach Juice. Biomolecules.

[31] Karthikeyan S., Kanimozhi G., Prasad N.R., Mahalakshmi R. (2011). Radiosensitizing effect of ferulic acid on human cervical carcinoma cells in vitro. Toxicol. In Vitro.

[32] Van Vuuren S., Viljoen A. (2011). Plant-based antimicrobial studies—Methods and approaches to study the interaction between natural products. Planta Med..

